# Growth and regrowth of adult sea urchin spines involve hydrated and anhydrous amorphous calcium carbonate precursors

**DOI:** 10.1016/j.yjsbx.2019.100004

**Published:** 2019-02-08

**Authors:** Marie Albéric, Cayla A. Stifler, Zhaoyong Zou, Chang-Yu Sun, Christopher E. Killian, Sergio Valencia, Mohamad-Assaad Mawass, Luca Bertinetti, Pupa U.P.A. Gilbert, Yael Politi

**Affiliations:** a Department of Biomaterials, Max Planck Institute of Colloids and Interfaces, 14424 Potsdam, Germany; b Department of Physics, University of Wisconsin, Madison, WI 53706, USA; c State Key Laboratory of Advanced Technology for Materials Synthesis and Processing, Wuhan University of Technology, 430070 Wuhan, China; d Materials Science Program, University of Wisconsin, Madison, WI 53706, USA; e Department of Molecular and Cell Biology, University of California, Berkeley, CA 94720, USA; f Helmholtz-Zentrum Berlin für Materialen & Energie, 12489 Berlin, Germany; g Departments of Chemistry, Geoscience, Materials Science and Engineering, University of Wisconsin, Madison, WI 53706, USA

**Keywords:** Sea urchin spine regeneration, Anhydrous amorphous calcium carbonate, Hydrated amorphous calcium carbonate, Ca *L*_2,3_-edge spectra, PhotoEmission Electron spectroMicroscopy

## Abstract

In various mineralizing marine organisms, calcite or aragonite crystals form through the initial deposition of amorphous calcium carbonate (ACC) phases with different hydration levels. Using X-ray PhotoEmission Electron spectroMicroscopy (X-PEEM), ACCs with varied spectroscopic signatures were previously identified. In particular, ACC type I and II were recognized in embryonic sea urchin spicules. ACC type I was assigned to hydrated ACC based on spectral similarity with synthetic hydrated ACC. However, the identity of ACC type II has never been unequivocally determined experimentally. In the present study we show that synthetic anhydrous ACC and ACC type II identified here in sea urchin spines, have similar Ca *L*_2,3_-edge spectra. Moreover, using X-PEEM chemical mapping, we revealed the presence of ACC-H_2_O and anhydrous ACC in growing stereom and septa regions of sea urchin spines, supporting their role as precursor phases in both structures. However, the distribution and the abundance of the two ACC phases differ substantially between the two growing structures, suggesting a variation in the crystal growth mechanism; in particular, ACC dehydration, in the two-step reaction ACC-H_2_O → ACC → calcite, presents different kinetics, which are proposed to be controlled biologically.

## 1 Introduction

**Fig. 1:**
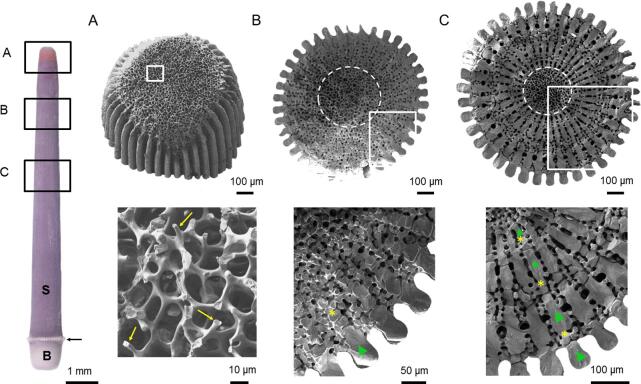
Morphology of the skeletal part of the *S. purpuratus* spines observed by SEM on transverse sections at different heights in the spine, after removal of the soft tissues. A) Growing tip showing broken micro-spines (yellow arrows) that form the inner stereom, B) section close to the tip where one septa layer (green triangle) and one thickened stereom layer (yellow star) are observed and C) section in the middle where 4 alternating layers of septa and thickened stereom are observed. The inner stereom is indicated by a dashed line circle in B) and C). The shaft of the spine (S), the base (B) by which the spine attaches to the tubercle of the body shell (the test), and the milled ring (arrow) are indicated on the overview of the spine in A). White boxes indicate where the higher magnification SEM images on the right were acquired. (For interpretation of the references to color in this figure legend, the reader is referred to the web version of this article.)

Adult sea urchins possess mineralized appendages called spines, which project from the test (the body shell) and are used for defense and locomotion. The mineralized structure of the spines is composed of calcite, small amounts of stable amorphous calcium carbonate (ACC), water, and intra-crystalline organic molecules (Aizenberg et al., 1997, Albéric et al., 2018b, Ameye et al., 2001, Berman et al., 1990, Kanold et al., 2015, Seto et al., 2012). The skeletal portion of the spines consists of an inner meshwork (stereom) and radial outer dense wedges termed septa (Fig. 1). The spines are covered and filled with soft tissues, namely the epidermis and the dermis (or stroma), which contain various cell types located all along the spine (Dubois and Ameye, 2001, Heatfield and Travis, 1975b, Heatfield and Travis, 1975a, Märkel and Röser, 1983, Märkel et al., 1989).

In protecting the test, spines serve as sacrificial appendages that constantly break and regenerate during the lifetime of sea urchins (Dubois and Ameye, 2001). The spines regenerate rapidly after fracture; with growth rates of 125 µm/day in length and 1 µm/day in width for the *Paracentrotus lividus* species (Gorzelak et al., 2011), and 160 µm/day in length for the *Strongylocentrotus purpuratus* (Heatfield, 1971c).

The regeneration of spines is a phenomenon particularly well-suited to study biomineralization in adult sea urchins since it has been described to be similar to their initial growth (Dubois and Ameye, 2001). Previous studies on sea urchin spine regeneration addressed the morphology of the growing mineralized microstructure and the involved cellular activity (Gorzelak et al., 2011, Heatfield, 1971c, Heatfield, 1971a, Heatfield and Travis, 1975b, Heatfield and Travis, 1975a, Märkel and Röser, 1983, Märkel et al., 1989). After sea urchin spines fracture, increased cell proliferation within the spines occurs in the milled ring (Heatfield, 1971a) (the location of the milled ring is indicated in Fig. 1). The proliferating cells migrate toward the fractured region where they fuse together to form a syncytium in which the regeneration starts (Märkel and Röser, 1983, Märkel et al., 1989). The inner stereom grows in the form of longitudinal micro-spines, which further connect transversally through bridges to form the final meshwork (Fig. 1A). The radial wedges are formed later by transversal growth (or thickening) of the outer part of the inner stereom to form the “thickened stereom”, which is progressively filled to form a septum (Fig. 1B). More stereom is then added to the exterior of each septum and this new stereom layer will then also thicken and be filled to become a layer of septa, *etc.* (Fig. 1C) (Heatfield, 1971b). The formation of the radial wedges is species specific: in *Strongylocentrotus purpuratus*, parts of the thickened stereom do not become septa, leading to the appearance of radial wedges consisting of alternating septa and thickened stereom (Fig. 1C), whereas in *Paracentrotus lividus* the septa are continuous. Two patterns of skeletal growth were distinguished during the regeneration/growth of sea urchin spines: 1) the formation of the inner stereom via the growth of the micro-spines and 2) the formation of the radial wedges via the thickening of the stereom and the partial (*S. purpuratus*) or total (*P. lividus*) filling of the stereom pores to form the septa (Heatfield, 1971b).

At the nano-scale, spine calcite mineral was shown to be a space-filling crystal as determined by surface adsorption analysis (Yang et al., 2011). The mineral phase involved in the initial growth of the *P. lividus* micro-spines was identified as an ACC phase, which later crystallizes into calcite in the living animal or when isolated spines are exposed to ambient conditions (Politi et al., 2004). The presence of ACC as an amorphous precursor was first observed in the biomineralization of sea urchin embryonic spicules (Beniash et al., 1999, Beniash et al., 1997, Raz et al., 2003) and subsequently discovered in many other mineralizing tissues from echinodermata as well as from various phyla e.g. mollusca, brachiopoda, cnidaria, porifera and crustacea (Addadi et al., 2003, Bahn et al., 2017, Baronnet et al., 2008, Cuif et al., 2008, Cusack et al., 2008, DeVol et al., 2015, Gilis et al., 2011, Goetz et al., 2011, Griesshaber et al., 2009, Jacob et al., 2008, Khalifa et al., 2018, Killian et al., 2009, Ma et al., 2007, Mass et al., 2017, Neues et al., 2011, Nouet et al., 2012, Politi et al., 2004, Przenioslo et al., 2008, Rollion-Bard et al., 2010, Von Euw et al., 2017, Weiss et al., 2002, Xiang et al., 2014). In addition, the study of biomineralization in sea urchin embryonic spicules has recently provided important insights into the ion transport mechanisms involved in mineralized tissue formation (Killian and Wilt, 2017, Vidavsky et al., 2014, Vidavsky et al., 2016, Vidavsky et al., 2015, Wilt, 2002). Whereas stabilized ACC in many organisms was identified as hydrated ACC (with 1:1 ratio of CaCO_3_:H_2_O), transient ACC in embryonic sea urchin spicules was identified to be predominantly anhydrous (Addadi et al., 2003, Raz et al., 2003). Moreover, analyses of high-resolution maps acquired from X-ray absorption near-edge (XANES) spectra generated from PhotoElectron Emission spectroMicroscopy (X-PEEM), revealed ACCs with two different spectral signatures (Gong et al., 2012, Politi et al., 2008). They were denoted by ACC type I and ACC type II. The Ca *L*_2,3_-edge spectra of the two ACC types showed distinct line shapes originating from changes in the relative position and intensity of the *L_3_* and *L_2_* crystal field splitting peaks, which are well resolved and sharper in the higher symmetry structure of calcite. ACC type I could be identified as hydrated ACC based on the similarity of its Ca *L*_2,3_-edge spectrum to that of synthetic hydrated ACC (Politi et al., 2008), whereas ACC type II was assigned to be anhydrous based on its dominant abundance in the growing spicule (Raz et al., 2003), *albeit* the Ca *L*_2,3_-edge spectrum of an anhydrous synthetic ACC has never been reported. Following these observations, a two-step reaction (ACC-H_2_O → ACC → calcite) for the formation of calcitic spicule in embryonic sea urchins was proposed (Gong et al., 2012, Politi et al., 2008).

Moreover, mostly anhydrous ACC was identified in fully developed adult sea urchin spines, once powdered and analyzed by thermogravimetric analyses coupled with differential scanning calorimetry (Albéric et al., 2018b). Anhydrous ACC was suggested to be a remnant phase, kinetically trapped during a two-step reaction similar to the one proposed for embryonic spicules. However, the spatial distribution of this remnant ACC remains uncertain, and may vary in the different structures, namely stereom and septa. Indeed, stereom and septa formation were suggested to occur through different crystal growth mechanisms based on anisotropy variation in the crystal structure (Aizenberg et al., 1997) and the presence of different intra-crystalline organic molecules (Ameye et al., 2001). It was also proposed that, in contrast to the stereom, septa formation might exclude ACC precursor phases and occurs via direct precipitation of calcite (Moureaux et al., 2010). In order to test these hypotheses, here we studied the spatial distribution of ACC phases in freshly re-grown micro-spines and older portions of stereom and septa using X-PEEM with high spatial resolution.

## 2 Materials and methods

### 2.1 Biogenic samples and sample preparation for X-PEEM experiments

**Fig. 2:**
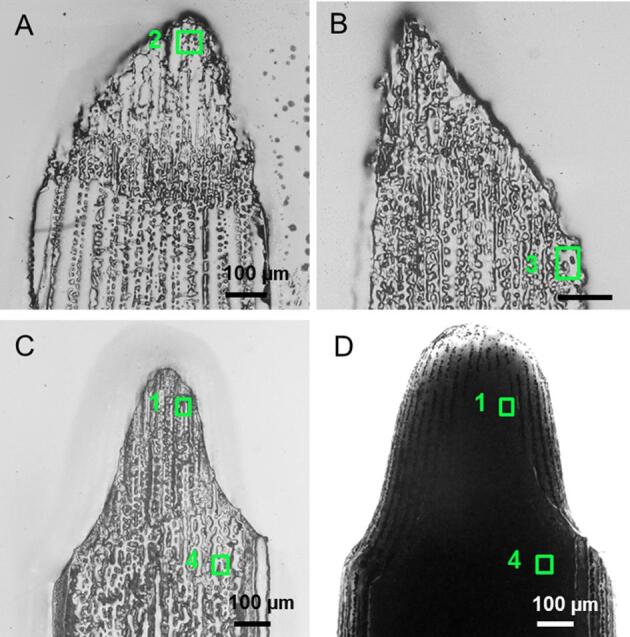
Four regions of interest measured by X-PEEM in the longitudinal polished sections. Optical images of the samples observed in transmitted and reflected light (left and right image in each set, respectively). A) Spine showing region 1 in the micro-spines and region 4 in the thickened stereom below the fracture plane, spine analyzed 27 h post mortem, note that soft tissues are conserved and observed in the transmitted light image. B) Spine showing region 2 in the micro-spines, spine analyzed 18 h post mortem and C) spine showing region 3 in the thickening stereom, spine analyzed 27 h post mortem.

Adult *Strongylocentrotus purpuratus* sea urchins were collected from intertidal and subtidal areas of the Pacific coast of Sonoma and Mendocino Counties of California by the Water Resources Group staff of the Bodega Marine Laboratory, University of California, Davis, (Bodega Bay, California, USA). The sea urchins were then transported and kept in refrigerated seawater (15 °C) tanks at the University of California, Berkeley (Berkeley, California, USA). Regenerating spines were cut off from the animals after selection based on macroscopic morphological criteria: the regenerating tip 1) had a smaller diameter than the shaft with a clear change in diameter just above the fracture plane and 2) was lighter in color than the shaft (pink vs. purple) (Fig. 1 and Fig. 2).

Before embedding the samples, two fixation methods of the soft tissues around and inside the spines were performed. One established fixation method successfully used in the case of growing corals (Mass et al., 2017) was tested. It involves the fixation of the samples in 2% paraformaldehyde and 0.05 M sodium cacodylate buffer in 22 g/L Na_2_CO_3_ for 50 min and dehydration steps of 5 min (ethanol/Na_2_CO_3_/cacodylate, from 50% to 100% ethanol in 7 steps). However, in the case of the sea urchin spines, the soft tissues were not well preserved after such fixation. Those samples were nevertheless embedded in a single 1″ round mold, polished down to 50 nm with alumina paste (Buehler, Lake Bluff, IL, USA), previously dialyzed against 22 g/L Na_2_CO_3_ for 24 h to prevent dissolution of ACC. They were then trimmed, cleaned, coated with 1 nm Pt in the area of interest and 40 nm Pt around it, as described in (De Stasio et al., 2003). Spines were embedded longitudinally in order to obtain an overview of the growth process, which is faster in the longitudinal direction. Moreover, polished sections enable observations and measurements of not only the surfaces of the mineralized tissues (micro-spines/stereom/septa) but also their core. We present here the X-PEEM results of a micro-spines (region2) and a thickening stereom (region 3) regions of two different spines (Fig. 2A and B). In the second fixation method used, the samples were simply immersed in anhydrous ethanol for 1 h. The rapid and direct fixation by ethanol allowed a better preservation of the tissues in the case of our samples (regions 1 and 4, Fig. 2C and D). Samples were then embedded and polished in the same orientation and with the same procedure as described above. For this second fixation method, we report the X-PEEM results for samples measured in regions containing micro-spines (region 1) and thickened stereom (region 4) (Fig. 2C and D).

Finally, depending on the polishing time needed to obtain suitable surfaces for X-PEEM measurements, samples were analyzed with post-mortem time ranging from 18 to 27 h. The time between cutting of the spines from the animal and X-PEEM analysis (post-mortem time) as well as the sample preparation may accelerate the amorphous phase transformations that would normally occur in the living animal. Therefore, if the two-step reaction (ACC-H_2_O → ACC → calcite) occurs in sea urchin spines, it may be at a more advanced stage in the analyzed samples than in the animal at the time of spine cutting. However, the careful methodology used here and developed previously has proved to minimize ACC transformation (Gong et al., 2012, DeVol et al., 2015, Killian et al., 2009, Mass et al., 2017). Moreover, similar regions (micro-spines) of samples analyzed 18 and 27 h post mortem were found to contain similar amount of ACC, with similar ratios of ACC and ACC-H_2_O for a specific growing stage (see section 3: results and discussion).

Additionally, in order to relate the present results to previously published work on bulk powdered spines (Albéric et al., 2018b), regions in fully developed *P. lividus* spines were measured. The *P. lividus* sea urchins were collected from Roscoff (Britanny, France, Atlantic Ocean) and housed in a seawater aquarium at the Max Planck Institute of Colloids and Interfaces (Potsdam, Germany). For both species, spines were cut off from the urchins and all soft tissues were removed by mild NaClO treatment (5%). After embedding, transverse sections (perpendicular to the long axis of the spine) were obtained in the middle of the spine.

### 2.2 Synthetic samples

ACC samples were synthesized by adding 2 mL of 1 M CaCl_2_ solution to 48 mL Na_2_CO_3_ solution using a controlled titration set up as in (Zou et al., 2015). The concentration ratio of calcium and carbonate after mixing was 1:1 and the pH of the solution changed from 11.4 to 10.6 from beginning to the end of the reaction. The ACC synthesis was followed by a fast filtration and drying procedure using cold ethanol (4 °C, 100%). The samples were stored in a vacuum desiccator for further use. Anhydrous ACC was obtained by mild heat treatment at 200 °C for 2 h. Fourier transform infrared (FTIR) spectroscopy was performed to verify the anhydrous nature of the annealed ACC sample. The FTIR spectra were recorded using a Thermo Scientific Nicolet iS5 FTIR spectrometer (ATR-Diamond mode) (32 scans, resolution 2 cm^−1^). The FTIR spectra of the hydrated ACC phase before and after annealing at 200 °C for 2 h are reported in Fig. A1, Appendix A. The spectrum of the annealed sample attests to the absence of water and validates its anhydrous nature as it also shows the characteristic features of an ACC phase (Fig. A1). The annealed phase is therefore called synACC hereafter and the hydrated phase is named synACC-H_2_O.

### 2.3 Scanning electron microscopy observations

The spines were treated with mild NaClO (5%) to remove the organic soft tissues and were fractured perpendicular to their long axis. Coated samples (∼5–10 nm Pt) were investigated using a scanning electron microscope (JEOL, JSM‐7500F) at acceleration voltage of 5.0 kV.

### 2.4 X-ray PhotoEmission Electron spectroMicroscopy experiments and data analysis

#### 2.4.1 Experimental setups

X-ray PhotoEmission Electron spectroMicroscopy (X-PEEM) experiments on biogenic samples were performed using the PEEM-3 microscope on beamline 11.0.1.1 at the Advanced Light Source (ALS) (Chopdekar, 2018), Lawrence Berkeley National Laboratory. The illuminating X-rays were circularly polarized, the sample voltage was −18 kV and the experiment was performed under ultra-high vacuum. For calcium component mapping, stacks consisting of absorption images at a specific photon energy were acquired while scanning the photon energy across the Ca *L_2,3_*-edge, from 340 to 360 eV, with steps of 0.1 eV between 345 and 355 eV, and steps of 0.5 eV elsewhere. Therefore, each pixel in a stack contains the full Ca *L_2,3_*-edge spectrum. The total integration time per energy was either 0.4 s or 1 s. The fields of view of the stacks were set to 55 and 60 μm with 55 and 60-nm pixels, respectively. All stacks were drift-corrected with the PEEM Vision software (Scholl, 2014).

The synthetic samples were measured with an Elmitec PEEM III microscope at the UE49_PGMa beamline at BESSY II, the synchrotron radiation source of the Helmholtz Zentrum Berlin (Germany) (Kronast and Valencia Molina, 2016). Incoming radiation was linearly polarized (horizontal) and illuminated the sample at 16° from its surface plane. Sample voltage was −10 kV and the experiment was performed under ultra-high vacuum. Stacks were obtained across the Ca *L*_2,3_-edge (340 eV-360 eV) in 0.1 eV steps. The total integration time per energy was 0.5 s. The field of view was set to 50 μm. The stacks did not require drift-correction.

#### 2.4.2 Data analysis

**Fig. 3:**
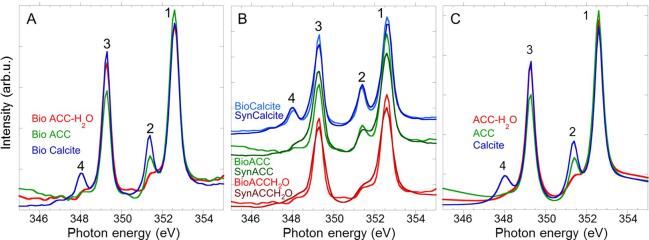
Ca *L*_2,3_-edge XANES spectra of A) biogenic and B) biogenic and synthetic calcite, ACC, and ACC-H_2_O. Ca *L*_2,3_-edge spectrum of SynCalcite is from (Gong et al., 2012). Each spectrum was fitted and the parameters of the fits (peak position and width) are reported in Table A1. C) The component spectra used to produce component maps are peak-fitted versions of the spectra in A).

We obtained a set of biogenic reference spectra termed “component spectra”, directly from sea urchin spines, presented in Fig. 3C, to be used for “component mapping”. In these reference spectra, the biogenic spectrum for each phase is an average of 60 spectra, each of which originates from a single pixel of 60 × 60 nm^2^ from about 15 different data sets (stacks) using Igor Pro® version 6.37 and the GG Macros 2019 that run in Igor. Pixels were selected if they were composed of over 90% ACC-H_2_O, ACC, or calcite respectively (as obtained using (Gong et al., 2012) reference spectra for ACC-H_2_O, ACC, and calcite).

All 60 single-pixel spectra for each phase were aligned to one another between 340 and 360 eV, i.e. rescaled in intensity and shifted in energy so that the peak intensities were on the same scale and all Ca L_2_-edge peaks 1 were at 352.6 eV. The 60 spectra were then aligned to and divided by a linear background calculated from a line fit to the pre-edge, allowing the rescaling but not the shifting of an individual spectrum. The 60 spectra were averaged, and the average ACC-H_2_O, ACC, and calcite spectra were aligned to one another between 340 and 360 eV firstly and between 345 eV and 355 eV secondly, retaining the linear background. These spectra were then fitted with four Lorentzian functions, two arctangent functions and one polynomial background (Fig. 3C). The fitted spectra were used as “component spectra” for all component maps presented here. For presentation purposes, all spectra in Fig. 3 were normalized to aliner background. For component mapping, instead, the component spectra remained lying on a steeply sloped linear background.

“Component maps” were obtained using the GG Macros 2019 by finding the best fit of each pixel’s spectrum to a linear combination of component spectra, and then displaying the fraction of each component as color in RGB maps where biogenic ACC-H_2_O is red, ACC is green, and calcite is blue (Fig. A2). The goodness of the fit is described by χ^2^ = (1/area under the background subtracted spectra)* $\sum \frac{{\left(data-fitted\right)}^{2}}{\mathrm{fitted}},$ where ‘data’ is the spectral intensity value as measured in PEEM and ‘fitted’ is the intensity based on the optimized linear combination of the three component spectra at each corresponding energy. The χ^2^ maps corresponding to each of the component maps are reported in Fig. A2.

The pixel size in the X-PEEM measurement was set to 55–60 nm. This size corresponds roughly to the nano-particles of ACC extracted from regenerating sea urchin micro-spines (Politi et al., 2004). Therefore, each pixel of the PEEM component maps is likely composed of more than one nanoparticle that might be of different phases. Pixels with mixed phases have mixed colors, such as yellow (mixture of ACC and ACC-H_2_O), cyan (calcite and ACC), and magenta (calcite and ACC-H_2_O). The maps were masked using a qualitative calcium concentration map created by dividing the average of 7 on-peak images acquired around 352.6 eV (peak 1) by the average of 9 images acquired in the pre-edge, around 345 eV. The Ca concentration map was leveled in Adobe Photoshop CC 2017® and the Magic Wand tool was used to select the brightest pixels in the image and to exclude everything else. Bright pixels that were clearly not part of the spine were masked off by hand.

The spectra for synthetic ACC-H_2_O and ACC were acquired from several measurements. For each measurement, individual spectra were extracted by binning 40 pixels to reduce the noise, they were then aligned, i.e. shifted in energy and rescaled in intensity as described above for the spectra of biogenic samples and finally averaged. The average spectra of each measurement were again aligned and averaged to obtain representative spectra for each ACC phases, which were then normalized and fitted as describe above. The synthetic calcite spectrum from (Gong et al., 2012) was used.

## 3 Results and discussion

### 3.1 Morphology of sea urchin spines at the different growth stages

The micro-architecture of the skeleton of fully developed and regenerated sea urchin spines has been described thoroughly for a number of species (Drozdov et al., 2016, Dubois and Ameye, 2001, Gorzelak et al., 2011, Heatfield, 1971c, Vinnikova and Drozdov, 2011). Following from these previous studies, we decided to limit the portions of the adult spine we examined with X-PEEM analyses, to the micro-spines, the thickening stereom, the thickened stereom and the septa of the *S. purpuratus* sea urchin (Fig. 2). After removal of the soft tissues, the growing micro-spines are seen in Fig. 1A, although, most of them are broken due to their fragility. The micro-spines (yellow arrows in Fig. 1A) are 2–4 µm wide and 5–8 µm long. Struts or bridges that connect the micro-spines are clearly seen and are about 3 µm wide. The micro-spines and the struts together form the inner stereom consisting of a porous meshwork. Regions selected from the regenerated *S. purpuratus* micro-spines measured by X-PEEM are presented: region 1 of a spine showing the soft tissue and region 2 of a spine whose soft tissue was lost during sample preparation (see Material and Methods) (Fig. 2).

The inner stereom and the outer radial wedges are observed on transverse fractured sections perpendicular to the long axis of the spine (Fig. 1 and Fig. A3). As a result of their formation pathway described in the introduction, the radial wedges of the *S. purpuratus* species consist of alternating layers of septa and thickened stereom. The thickened stereom differs from the inner stereom as the former presents trabecular struts ∼10 µm wide whereas the later contains thinner struts ∼5 µm wide when observed in transverse sections (Fig. 1B). This alternation of septa and thickened stereom leads to the appearance of concentric layers. The number of concentric layers increases from the distal part (the tip) of the spine where only one layer is present to the proximal part (near the milled ring) where up to seven layers can be observed (Fig. 1 and Fig. A3). Close to the milled ring, where the spine is the oldest, the two outermost septa fuse as a result of pore filling in the thickened stereom (Fig. A3).

Two analyzed stereom regions are reported: one is called “thickening stereom” (region 3) and the other is named “thickened stereom” (region 4) (Fig. 2). The “thickening stereom” was likely still actively thickening to become a septum at the time the spine was cut from the animal, as it is located at the exterior and close to the growing tip of the spine (Fig. 2). On the contrary, the thickened stereom region is located below the fracture plane and is part of the radial wedges described above. Region 4 is therefore likely older than region 3.

Finally, stereom and septa regions located in transverse sections originating from the middle of *S. purpuratus* and *P. lividus* fully developed spines were also measured. The main difference between the *S. purpuratus* and *P. lividus* species is the morphology of the radial wedges: in *P. lividus* there are only septa with no thickened stereom remaining, whereas in *S. purpuratus*, septa and thickened stereom layers alternate and persist.

### 3.2 ACC and ACC-H_2_O spatial distribution in growing sea urchin spines

Amorphous calcium carbonate phases were already identified in adult sea urchin spines, in particular, in freshly regenerated micro-spines (Politi et al., 2004) and in bulk fully developed spines (Albéric et al., 2018b, Seto et al., 2012). Here, we show evidence that *S. purpuratus* sea urchin spines exhibit three different Ca *L*_2,3_-edge spectra (Fig. 3A), which are very similar to the ones previously measured in sea urchin embryonic spicules (Gong et al., 2012, Politi et al., 2008), supporting the presence of the same three calcium carbonate phases in embryonic spicules and adult spines, namely ACC type I and II, and calcite.

As previously reported, the Ca *L*_2,3_-edge spectrum of calcite shows the characteristic four distinct peaks corresponding to the crystal field split of the *L*_2_ and *L*_3_ lines (de Groot et al., 1994, Ko et al., 2007). The peaks are numbered 1–4 for consistency with previous publications, with peak 1 and 2 related to the highest energy *L*_2_ white lines and peaks 3 and 4 to the lowest energy *L*_3_ white lines. Although peaks 1 and 3 are similar in line shape in the Ca *L*_2,3_-edge spectra from the amorphous phases and calcite, peaks 2 and 4 show variability across the three materials (Fig. 3A). Both ACC type I and II spectra show a shoulder at the position of peak 4, but ACC type II has a well-resolved and sharp peak 2, whereas the ACC type I spectrum only shows a shoulder at the approximate position of peak 2 (Fig. 3A). The observed variations have been related, by modeling, to varying levels of disorder of the carbonate ions in the two structures relative to calcite (Rez and Blackwell, 2011), however the effect of hydration on the Ca *L*_2,3_-edge spectra of ACC still needs testing. Nevertheless, ACC type I was identified as hydrated ACC (DeVol et al., 2015, Gong et al., 2012, Killian et al., 2009, Mass et al., 2017, Politi et al., 2008), as the biogenic spectrum was similar to the synthetic ACC-H_2_O spectrum (Politi et al., 2008). ACC type II was suggested to be anhydrous ACC based on previous TGA-DSC measurement (Raz et al., 2003), which showed that spicules were mostly anhydrous. However, at the time of this study, synthetic anhydrous ACC had not yet been prepared successfully and could not be directly compared spectroscopically with its biogenic counterpart. The assignment of ACC type II to anhydrous ACC, at that time, was a reasonable assumption but not an experimentally proven point of fact.

Here, we report the Ca *L*_2,3_-edge spectra of synthetic anhydrous ACC (SynACC) as well as the ones of synthetic hydrated ACC (SynACC-H_2_O) and compare them to their biogenic analogs in growing sea urchin spines (BioACC and BioACC-H_2_O) (Fig. 3B, Table A1 fit parameters). Synthetic calcite is also reported from previous publication (Gong et al., 2012) and is compared to the sea urchin spine calcite (Fig. 3B). The Ca *L*_2,3_-edge spectra for biogenic and synthetic components are very similar (Fig. 3B) confirming the assignments of ACC, ACC-H_2_O and calcite in sea urchin spines as well as in previously studied systems (DeVol et al., 2015, Gong et al., 2012, Killian et al., 2009, Mass et al., 2017, Politi et al., 2008). In particular, the Ca *L*_2,3_-edge spectrum of synthetic ACC presents one clear crystal field splitting peak, peak 2, and a shoulder at peak 4, as previously measured for presumed biogenic ACC. Despite the similarities, we also note slight differences between biogenic ACC originating from the adult sea urchins and synthetic ACC. In particular, peak 2 is slightly shifted in BioACC (351.40 eV versus 351.46 eV in SynACC) towards its position in calcite (351.37 eV), and it is narrower (0.23 versus 0.31 eV in SynACC) than in SynACC (Table A1). This suggests that BioACC is more ordered than SynACC, as theoretically predicted by (Rez and Blackwell, 2011), and is consistent with the experimental results of (Politi et al., 2006) (for sea urchin embryos) that showed increased short-range order of biogenic ACC.

**Fig. 4:**
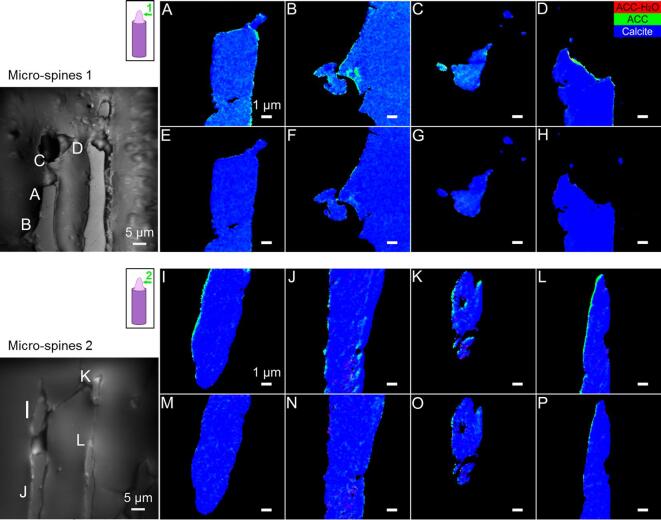
Component maps of selected areas of the measured X-PEEM regions 1 and 2 visualized via their average X-PEEM image (average intensity of all the images of the stack). Regions 1 and 2 are located in the growing micro-spine areas at the growing tips of two different spines indicated on the scheme by the green arrows. A-D) Region 1, spine analyzed 27 h post mortem. The color of each pixel corresponds to the proportion of ACC-H_2_O (red), ACC (green), and calcite (blue) at the pixel location. E-H) Repeat measurement of A-D. I-L) Region 2, spine analyzed 18 h post mortem and M-P) repeat measurement of I-L selected areas. Magenta or cyan pixels are displayed when the fitted spectrum can be described as a linear combination of calcite with either ACC-H_2_O or ACC, respectively. The scale bars correspond to 1 µm in all sub-figures. (For interpretation of the references to color in this figure legend, the reader is referred to the web version of this article.)

**Fig. 5:**
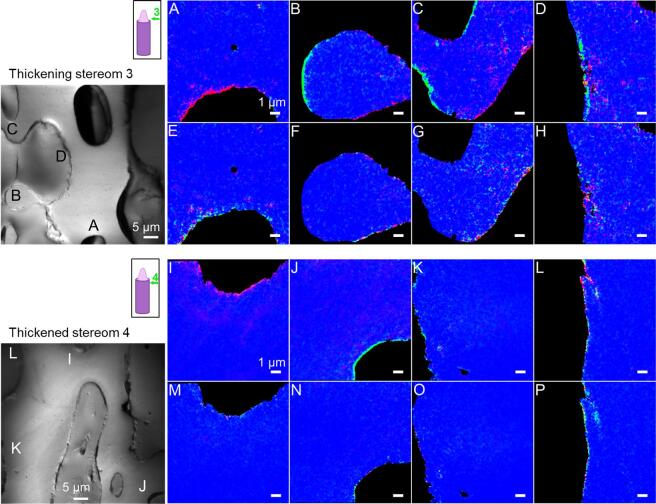
Component maps of selected areas of the measured X-PEEM region 3 and 4 visualized via their average X-PEEM image and respectively located in the thickening stereom and the thickened stereom as indicated by the green arrows on the scheme of the growing spine. Both regions were analyzed 27 h post mortem. A-D) Region 3, thickening stereom and E-F) repeat measurement of A-D selected areas. I-L) Region 4, thickened stereom and M-P) Repeat measurement of I-L selected areas. The color of each pixel corresponds to the proportion of ACC-H_2_O (red), ACC (green), and calcite (blue) at the pixel location. The scale bars correspond to 1 µm in all sub-figures. (For interpretation of the references to color in this figure legend, the reader is referred to the web version of this article.)

**Fig. 6:**
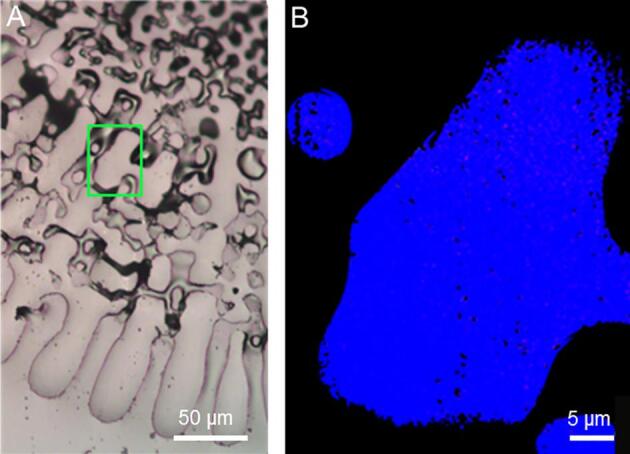
A) Optical image of a transverse section of a *S. purpuratus* fully developed spine, the analyzed septum is indicated by a green box. B) Component maps of the indicated septum. The region contains only calcite (blue pixels). The repeat measurement is reported in Fig. A4. (For interpretation of the references to color in this figure legend, the reader is referred to the web version of this article.)

To further elucidate the role of ACC-H_2_O and ACC in the biomineralization pathways of adult sea urchin spines, we first evaluated their amount in the different growth stages. Second, we determined their relative abundance and spatial distribution with the intention of addressing the mineral deposition and dehydration rates. To do so, X-PEEM “component maps” were obtained by fitting the spectrum of each pixel in the map to a linear combination of component spectra, that is, calcite, ACC, and ACC-H_2_O spectra (peak-fitted version, Fig. 3C) (see Material and Methods). Selected areas of the component maps as well as the entire maps are displayed in Fig. 4, Fig. 5 and Fig. A2, Fig. 6 and Fig. A4, respectively. The average Ca *L*_2,3_-edge spectra for each phase in regions 1–4 are presented in Fig. A5.

Generally, the amorphous phases (ACC and ACC-H_2_O) were more abundant in the micro-spines and the thickening stereom than in the thickened stereom and the septa (Fig. 4, Fig. 5, Fig. 66, A4). The amount of ACCs found decreased with the degree of maturation of the different regions of interest, which confirms that ACC-H_2_O and ACC are precursor phases involved in the mineral formation of adult sea urchin spines. These phases were previously observed on the surface (Politi et al., 2008) and in cross-sections (Gong et al., 2012) of fresh, forming sea urchin spicules of *S. purpuratus* suggesting that crystal growth in embryonic and adult systems involves similar mineral phases and phase transitions even though the microstructures of the two biominerals differ significantly.

Despite differences in the sample fixation and post-mortem time, the micro-spines regions (regions 1 and 2) present similar quantities and spatial distribution of ACCs (Fig. 4). The component maps show more ACC than ACC-H_2_O, suggesting that the transformation of ACC-H_2_O to ACC is relatively rapid, similar to the phase transition proposed for spicules (Gong et al., 2012, Politi et al., 2008). The anhydrous ACC phase is either distributed heterogeneously in the core of some of the micro-spines (region 1, Fig. 4A-C) or located densely at their surface (i.e. at the borders of the micro-spines in the longitudinally polished sections) (region 1, Fig. 4D and region 2, Fig. 4I and L). When distributed within the bulk/center of the micro-spines, both ACC and ACC-H_2_O coexist with calcite, as indicated in the maps by scattered clusters with cyan and magenta colors, respectively. From the comparison of the first and repeat measurement of the micro-spines regions (Fig. 4E-H and M-P), it appears that in the microscope chamber conditions, some ACC (green) and ACC-H_2_O (red) regions transform into calcite (blue) and some ACC-H_2_O regions transform into ACC. Both transformation sequences can be expected from thermodynamic and kinetic considerations, as a result of dehydration by evaporation under vacuum and radiation damage from exposure to the x-ray beam. We observed few exceptions to this trend, however, the spectra display high noise levels. In some rare cases, ACC-H_2_O or ACC regions remain untransformed after repeated measurements.

In the thickening stereom (region 3), likely on its course to become a septum in the living animal, both ACC-H_2_O and ACC were also identified (Fig. 5A–D). This suggests that the stereom (via micro-spines formation) and the septa (via stereom thickening) crystal growth might occur through similar amorphous precursor phases. Nevertheless, the comparison between regions 2 (Fig. 4I–L) and 3 (Fig. 5A–D) shows a higher abundance of ACC-H_2_O in the thickening stereom than in the growing micro-spines, suggesting that the dehydration is slower or less frequent in the case of the stereom thickening (septa formation). ACC-H_2_O and ACC are both located in the core and at the surfaces of the thickening stereom. Across the bulk of the stereom, Fig. 5C and D show scattered regions containing mixed phases, indicated by magenta pixels (calcite and ACC-H_2_O) and cyan pixels (ACC and calcite). Fig. 5A and B show rich ACC surfaces, consisting of either ACC-H_2_O or ACC suggesting that the thickening process occurs asymmetrically around the stereom.

In the thickened stereom (region 4), amorphous precursors were located predominantly at the surface and were identified as mixtures either of calcite and ACC-H_2_O (magenta pixels), of ACC-H_2_O and ACC (yellow pixels) or as single-phase ACC (green pixels) and ACC-H_2_O (red pixels) (Fig. 5I-L). It appears that the presumably older stereom (region 4) has indeed less ACCs than the newer stereom (region 3) but with a similar ACC/ACC-H_2_O ratio, i.e. more ACC-H_2_O than ACC. The presence of ACC-H_2_O in the thickened stereom was at first unexpected, since the stable amorphous calcium carbonate identified in the bulk spines is mostly anhydrous (Albéric et al., 2018b). However, only ACC phases occluded in the calcite crystals were measured in this TGA-DSC study (Albéric et al., 2018b) as the surface of the skeletal part was bleached and likely washed away with water. Therefore, the ACC-H_2_O phase observed at the surface of the thickened stereom by X-PEEM was likely not included in the TGA-DSC results.

The observation of surface ACC-H_2_O and ACC suggests that crystal growth still occurs at the surface of the thickened stereom, although at a slower rate. Indeed, as in the thickening stereom, ACC-H_2_O is more abundant than ACC, which, suggests that the mineral transformation is slower in the thickened stereom than in the micro-spines. These observations may indicate a biological control over the kinetic stability of ACC during various growth stages, which can in principle be achieved by additives (Addadi et al., 2003, Albéric et al., 2018a). Additionally, it was previously reported that specific cellular compartments (Heatfield and Travis, 1975b, Märkel and Röser, 1983) as well as specific intra-crystalline molecules (O-glycoproteins) (Ameye et al., 2001), localized at the surfaces of fully developed stereom are responsible for inhibiting (or slowing down) further mineralization. Additionally, variations in mineral transformation rate could in principle lead to differences in the structure of the final mineral phase, as observed previously (Aizenberg et al., 1997).

Finally, the regions (stereom and/or septum) in the fully developed spines of the *S. purpuratus* (Fig. 6) and *P. lividus* (Fig. A4) lack ACC. Previous works suggested that remnant ACC in fully developed spines is likely distributed in thin layers or inclusions of several nm associated with organics (Albéric et al., 2018b, Seto et al., 2012). It therefore appears that the spatial resolution in the present X-PEEM experiments (50–60 nm) is not sufficient to detect these ACC layers. Alternatively, exposure to vacuum or radiation damage crystallized these layers before they could be detected.

## 4 Conclusion

Our studies presented here demonstrate that synthetic and biogenic anhydrous ACC have spectroscopically similar signatures and, in turn, confirm the role of anhydrous transient ACC phases in various mineralizing organisms. Moreover, we identified ACC-H_2_O and ACC in adult sea urchin spines, especially in the growing micro-spines, the thickening and thickened stereom regions. The amount and distribution of these amorphous forms suggest that crystal growth of both stereom and septa involves similar amorphous precursor phases. Nevertheless, the mineral transformation in the septa, specifically the dehydration step appears to be slower than in the stereom. A quantitative understanding of how the sea urchin tunes the kinetic stability of the amorphous precursor phases in growing and regenerating spines will require further investigation.

## Declaration of interests

None.
