# Supplementary material for: Growth and regrowth of adult sea urchin spines involve hydrated and anhydrous amorphous calcium carbonate precursors
Source: J Struct Biol X. 2019 Feb 8;1:100004. doi: 10.1016/j.yjsbx.2019.100004 (PMC7337052; doi:10.1016/j.yjsbx.2019.100004)
Supplement: Supplementary data 1 [file mmc1.docx]

**Appendix**


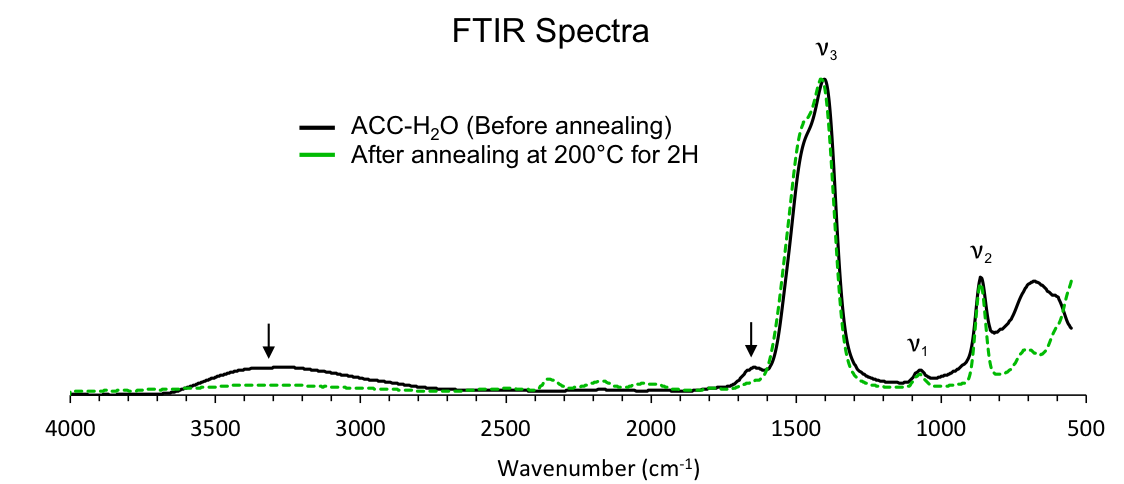


Figure A1. FTIR spectra of the synthetic hydrated ACC phases before (in black) and after (in green dashed line) mild annealing at 200°C for 2 hours. The black spectrum shows the characteristic peaks of hydrated ACC with a splitting in the asymmetric stretch of the carbonate ions (ν3) around 1470 and 1400 cm^-1^ and a broad peak around 660 cm^-1^. The peaks at 1070 and 860 cm^-1^ correspond to the symmetric stretch (ν1) and out-of-plane bending (ν2) bands of CO_3_^2-^, respectively. The broad bands around 3270 and 1620 cm^-1^ (black arrows) are due to vibrations of structural water in ACC. After annealing (green spectra), these broad bands are absent and all the others peaks characteristic of ACC are present confirming the nature of an anhydrous ACC phase. Note that the small peaks between 1900 and 2400 cm^-1^ observed in the green spectrum are due to CO_2_ from air.


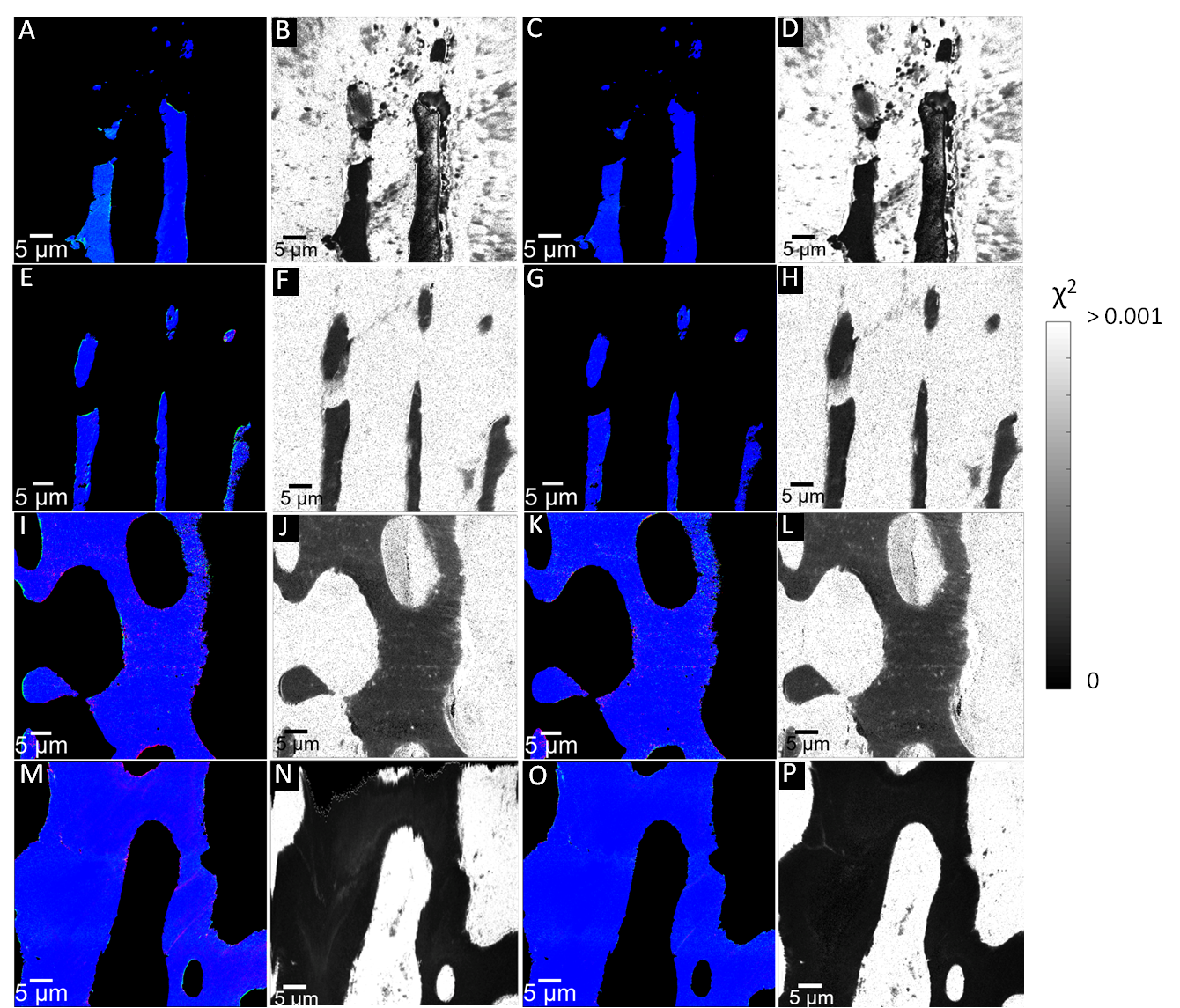


Figure A2. Measured X-PEEM areas visualized via their component maps where selected areas are presented in Figures 4 and 5. A) Component map, B) χ^2^ map of A), C) repeat component map of A) and D) χ^2^ map of C) of region 1. E-H), I-L), and M-P) show analogous information for the regions 2, 3, and 4, respectively. The χ^2^ parameter describes the goodness of the fit of each pixel’s spectrum to a linear combination of the component spectra. It is very low in the mineral areas indicating a good fit in those parts whereas it is higher in the epoxy parts where the level of noise of the spectra were too high to obtain a good fit.


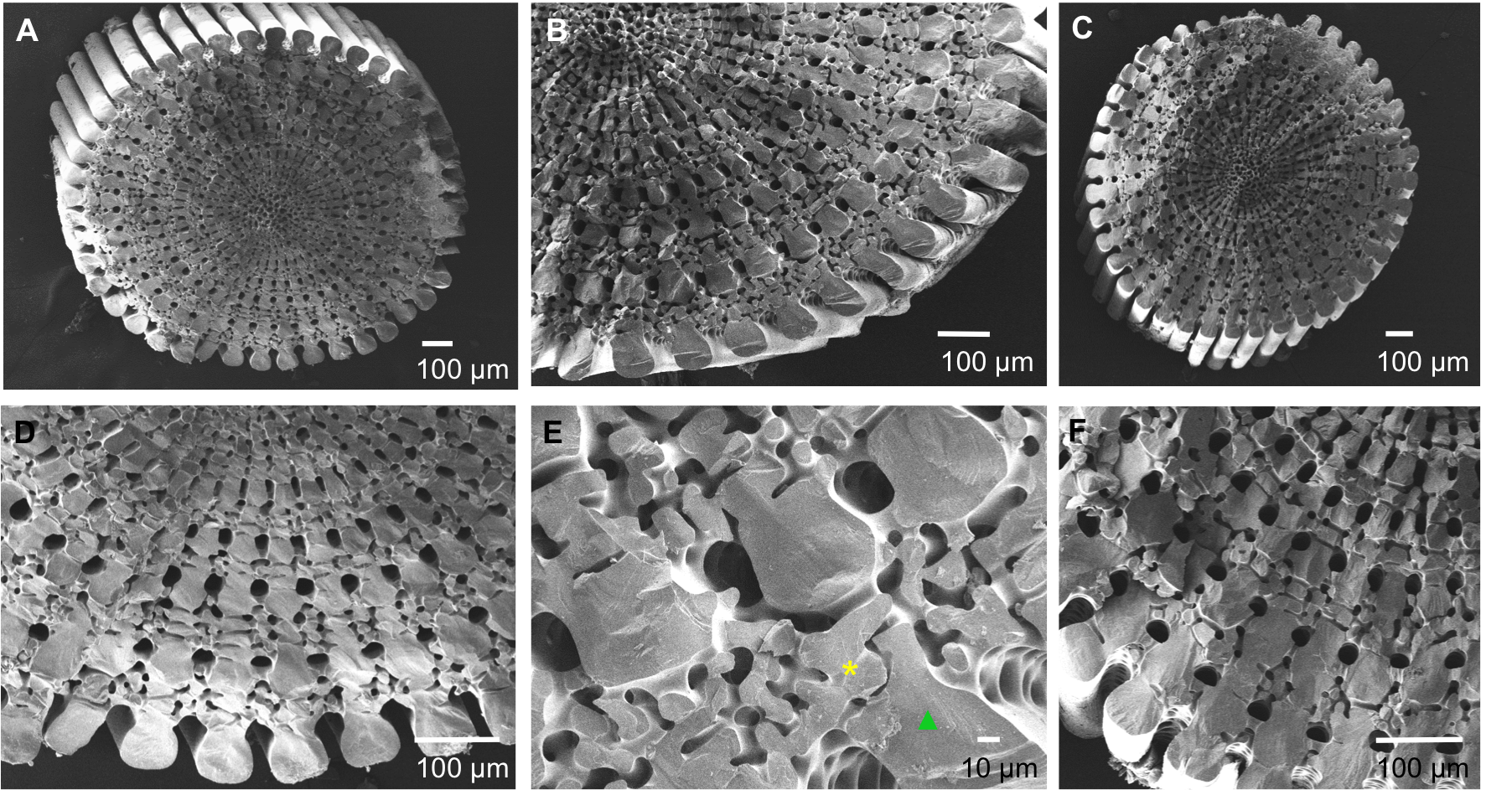


Figure A3. Morphology of the skeletal part of transverse sections (perpendicular to the long axis of the spine) of *Strongylocentrotus purpuratus* sea urchin spine close to the milled ring, A-C) overview of three different sections, D-F) higher magnification of the radial wedges of A-C). E) shows a septum (green triangle) and a thick stereom (yellow asterisk) about to fuse, and F) shows the two last external layers of septa that already fused.


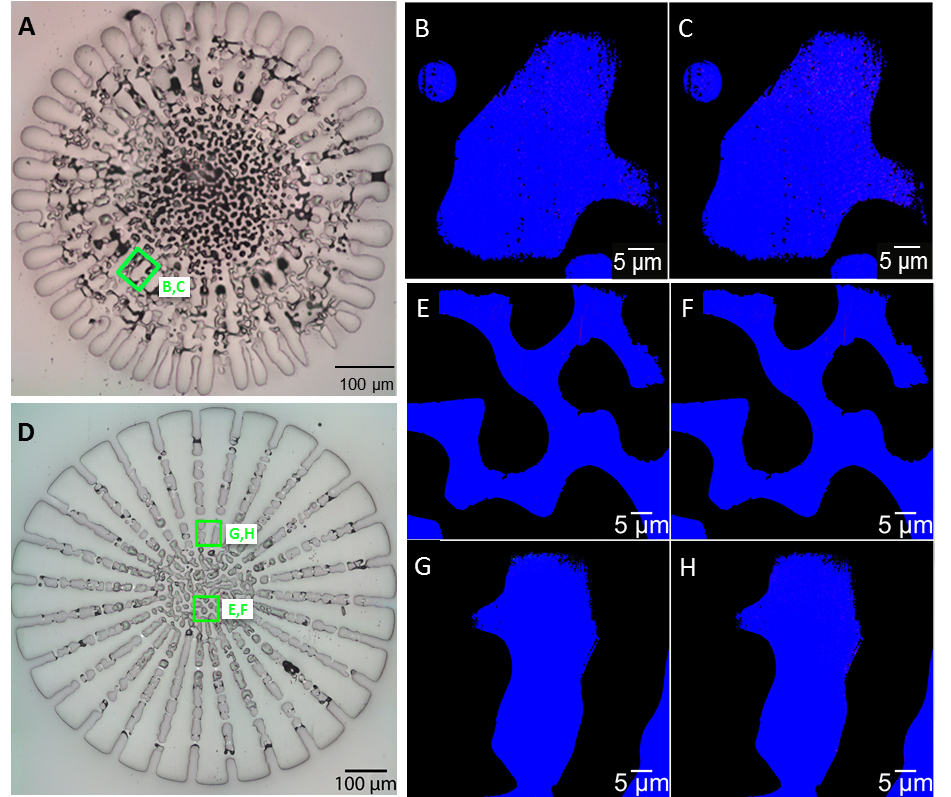


Figure A4. Component maps of fully developed spines cut perpendicular to their long axis of (A-C) *Strongylocentrotus purpuratus*: A) bright field optical image indicating the location of the septum component map B) and C) its repeat measurement. (D-H) *Paracentrotus lividus*: D) bright field optical image indicating the location of the stereom and septum component map E), G) and F), H) its repeat measurement, respectively.


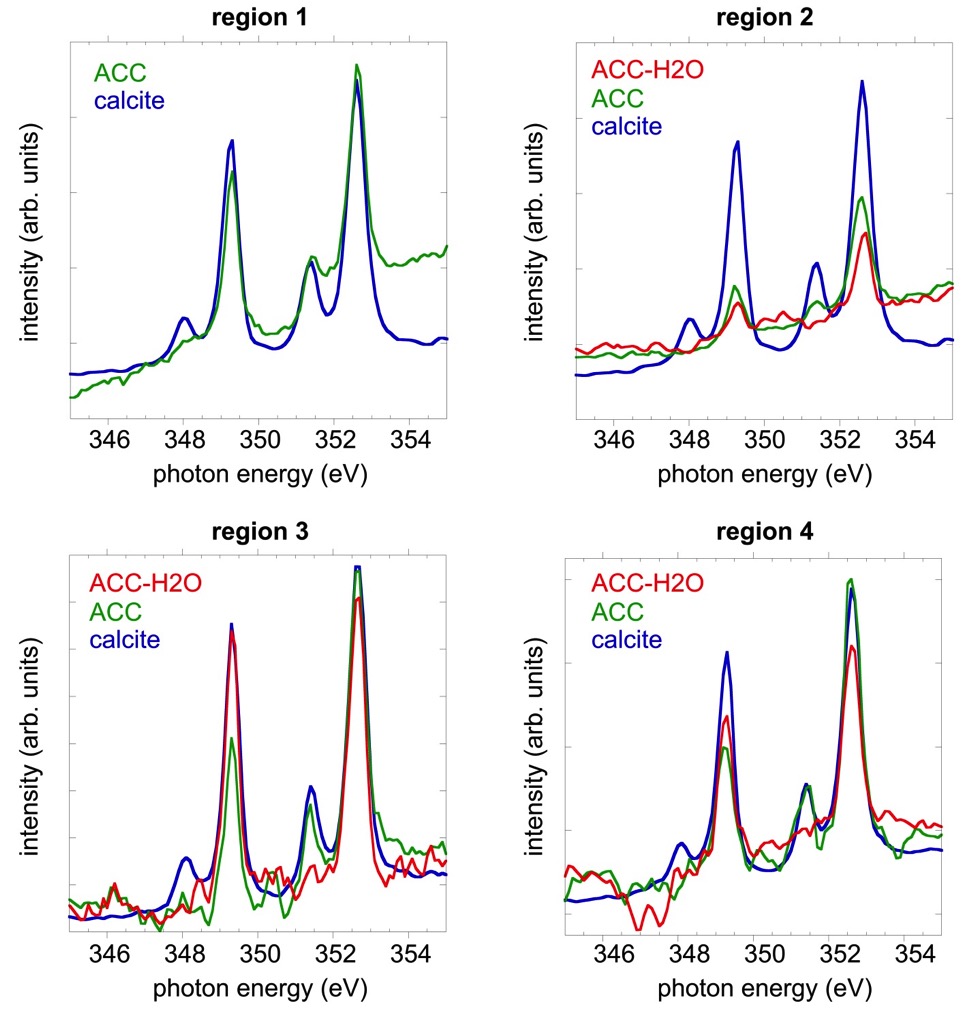


Figure A5. Extracted spectra of region 1 (micro-spines), region 2 (micro-spines), region 3 (thickening stereom) and region 4 (thickened stereom) for the three calcium carbonate phases: ACC-H_2_O (red), ACC (green), and calcite (blue). The spectra were obtained from averaging between 4-10 pixels per phase that were composed of over 90% ACC-H_2_O, ACC, or calcite, hence the spectra presented are noisy, like a single pixel spectrum.

Table A1. Peak fitting parameters of the experimental spectra presented in Figure 2B. The peak-fitted spectra used as component spectra for all component maps are presented in Figure 2C.

|  | Peak 1 | | | Peak 2 | | | Peak 3 | | | Peak 4 | | |
| --- | --- | --- | --- | --- | --- | --- | --- | --- | --- | --- | --- | --- |
|  | Amplitude | Position | Width | Amplitude | Position | Width | Amplitude | Position | Width | Amplitude | Position | Width |
| Biologic ACC-H_2_O | 0.72 | 352.59 | 0.52 | 0.20 | 351.44 | 1.02 | 0.55 | 349.27 | 0.49 | 0.04 | 348.2 | 1 |
| Biologic ACC | 0.86 | 352.59 | 0.56 | 0.23 | 351.4 | 0.62 | 0.45 | 349.27 | 0.5 | 0.04 | 348.19 | 1 |
| Biologic Calcite | 0.73 | 352.6 | 0.51 | 0.31 | 351.37 | 0.60 | 0.55 | 349.27 | 0.45 | 0.18 | 348.01 | 0.78 |
| Synthetic ACC-H_2_O | 0.92 | 352.57 | 0.73 | 0.31 | 351.47 | 1.09 | 0.72 | 349.27 | 0.69 | 0.35 | 348.04 | 2.59 |
| Synthetic ACC | 0.86 | 352.58 | 0.66 | 0.31 | 351.46 | 0.93 | 0.71 | 349.25 | 0.63 | 0.15 | 348.19 | 1 |
| Synthetic Calcite | 0.85 | 352.62 | 0.62 | 0.43 | 351.37 | 0.70 | 0.68 | 349.25 | 0.59 | 0.21 | 347.96 | 0.65 |
